# A biomechanical study of neck strength and impact dynamics on head and neck injury parameters

**DOI:** 10.3389/fbioe.2025.1597267

**Published:** 2025-09-03

**Authors:** Rahid Zaman, Mohammad Ibrahim Hossain, Ahmed Zubayer Raiyan, Shuvo Chowdhury, Aaron Jackson, Arthur Thomas Koster, Ashfaq Adnan

**Affiliations:** Department of Mechanical and Aerospace Engineering, University of Texas at Arlington, Arlington, TX, United States

**Keywords:** traumatic brain injury, neck injury, injury criteria, head impact, injury biomechanics, musculoskeletal modeling, neck strength, impact biomechanics

## Abstract

**Introduction:**

Head and neck injuries, including traumatic brain injuries (TBI), are a leading cause of disability and death worldwide. It affects millions of people worldwide, from automobiles to sports to military personnel. This study investigates the influence of impact locations, severities, and neck strength on head and neck injury parameters using a musculoskeletal head-neck model in OpenSim software.

**Methods:**

We hypothesize that eccentric impacts, particularly those on the anterolateral side, increase GAMBIT and Neck Injury Criteria (NIC) due to elevated rotational accelerations, and that higher neck strength mitigates GAMBIT and NIC under these impacts. To test our hypotheses, we investigated a total of 63 cases in which seven impact locations (two from the anterior side, two from the posterior side, and three from lateral sides), three neck strengths (low, mid, high strength capacity), and three impact severities (low, moderate, and high) were explored. Seven output parameters were analyzed: linear and rotational accelerations, the Generalized Acceleration Model for Brain Injury Threshold (GAMBIT), neck force, neck moment, and Neck Injury Criteria (NIC) and neck muscle strain.

**Results:**

Results reveal that anterolateral eccentric impacts pose the greatest risk, with rotational acceleration reaching 4,176 that is 4.75 times higher than anterior central impacts (879 rad/s^2^). GAMBIT values for moderate and high severity impacts are 1.44 and 1.54 times greater than low severity impacts, respectively. Head and neck injury parameters vary minimally (10) with neck strength.

**Discussion:**

In summary, the severities and location of impacts had a significant role in GAMBIT and NIC, and the anterolateral eccentric impact had a higher probability of head and neck injury than the other six impact locations. These findings underscore the critical role of impact location and severity in injury risk and suggest helmet padding in lateral and anterolateral zones with energy-absorbing materials to reduce rotational acceleration.

## 1 Introduction

An estimated 1.6 to 3.8 million sport and recreation-related traumatic brain injuries (TBIs) occur in the USA annually (Borich et al., 2013). Over 6 million passenger car accidents take place every year, and more than 42,514 people lost their lives in 2022 in the USA (Insurance Institute for Highway Safety and Highway Loss Data Institute, 2024). According to the CDC, in 2019, 60,611 TBI-related deaths occurred in the USA (Centers for Disease Control and Prevention, 2019). Along with the TBI, neck injuries are also common in high-energy events like blasts, automobile accidents, and sports impacts. It is reported that half a million people faced cervical spine injuries in high-energy accidents in the USA from 2005 to 2013, and the trend of incidents has increased from 4.1% to 5.4% (Johnson et al., 2020). A major portion of these head and neck injury events has been widely underreported. Even in fully equipped settings, like sports, 66% of TBI go unreported (McCrea et al., 2004). According to the Defense Health Agency, 505,896 service members faced mild to severe head and neck injuries from 2000 to 2024 (Defense Health Agency, 2024). In a study, it was found that 57% of service members who faced head and neck injuries did not seek medical care (Escolas et al., 2020). So, the percentage of the civil population that ignores head and neck injuries is really a concern. Moreover, the complications from TBI and neck injuries have long-term effects and can alter a person’s thinking, sensation, language, or emotions, which may not be immediately apparent. That’s why TBI and neck injuries are frequently referred to as a “silent epidemic” (Takhounts et al., 2011).

TBI is a complex pathophysiological process induced by mechanical loading of the brain. TBI can occur because of a direct impact or blow to the head or an indirect inertial response due to a non-cranial impact on the human body. Most of the life-threatening head and neck injuries from contact sports, automobiles, and blasts involve the torso, spine, and head. The rotational response of the head has been correlated to the risk of TBIs such as subdural hematomas and Diffuse Axonal Injury (DAI) (Browne et al., 2011). The effect of neck strength on concussions is still under investigation. Neck resistance and strength have an effect on the human head response during and after an impact (Farmer et al., 2022). Additionally, certain brain structures exhibit directional response dependencies, such as the falx and tentorium cerebri, particularly in the corpus callosum and brainstem, which undergo significant strain during axial rotations (Ho et al., 2017). Most studies indicate that stronger and stiffer necks reduce the risk of concussion (Fanta et al., 2013; Eckner et al., 2014; Hasegawa et al., 2014; Mang et al., 2015). However, some studies disagree with this (Mihalik et al., 2011). To gain a comprehensive understanding of the effects of neck strength and impact dynamics on head and neck injuries, three major parameters need to be carefully investigated. First, a computational or experimental head-neck model that can mimic head-neck response with high fidelity. Second, the input data for the analysis should be free from bias. Third, impacts from all around the head should be considered, as they can cause head and neck injuries.

A very detailed model of the human head and neck is a vital tool to assess any injury. The cervical spine is a pivotal structure of head dynamics and controls the head’s response after external loading. It establishes the mechanical linkage between the torso and head, and stabilizes the head using cervical disc and neck muscle tissue resistance. The most popular experimental and computational tools for reconstructing head and neck injury events are anthropomorphic test devices (ATD), multibody models, finite element models (FEM), and musculoskeletal models. Since all ATD use pin joints to connect the vertebrae, it allows limited rotation and bending in a single plane (Farmer et al., 2022). ATDs have 2-10 times stiffer necks and are mainly used for frontal, rear, and near-side automotive impact (Farmer et al., 2022). It lacks the natural compliance and multi-planar motion of human intervertebral discs and ligaments. ATDs can not mimic the intervertebral rotation with high biofidelity. Therefore, the evaluation of the rotational acceleration-based head injury criterion and Neck Injury Criterion (NIC) is not fully reliable with ATD experiments. Moreover, ATDs are expensive for repetitive blast and severe impact experiments. For these reasons, there is a growing interest in using fast and high bio-fidelity computational models. At the initial stages, lumped parameter models (Bumberger et al., 2025; Merrill et al., 1984; Deng and Goldsmith, 1987) and multibody models (Garcia and Ravani, 2003; Huo and Wu, 2017) of head and neck were developed. Most of those models are based on simplified rigid linkages. With the increase in computational efficiency, finite element models (FEM) became popular (Kleiven, 2007; Pramudita et al., 2009; Zhang et al., 2011; Gabler et al., 2019; Wu et al., 2019; Dymek et al., 2021; Liu et al., 2022; Putra et al., 2022; Singh et al., 2022; Tang et al., 2023; Wang et al., 2023). Finite element models were widely used to study the head and neck motion for whiplash (Zhang et al., 2011; Putra et al., 2022). However, FEM models are computationally very heavy and require predefined boundary conditions. Also, it is very difficult for the FEM model to exactly mimic the nature of muscle tissues and their interactions with the surrounding anatomical structures, such as muscle wrapping, dynamics, muscle excitations and contractions, and interactions with tendons and skeletons. The reliability of muscle force-generating capacity is a contributing factor in determining the head-neck model response. Not only does the shape and alignment of the cervical spine stabilize the head-neck joints, but the ligaments and voluntarily controlled muscles also play a contributing factor (Fanta et al., 2013). To understand the response of the head and neck under external loadings, it is essential to integrate all these physiological and biomechanical properties into the model to estimate a model’s muscle force-generating capacity. However, FEM model lacks those properties, hindering its ability to accurately simulate anatomical interactions.

Computational musculoskeletal models offer enhanced biofidelity compared to FEM and multibody models, as they incorporate dynamic inputs, thereby reducing reliance on static assumptions. These steps are critical for accurately capturing the biomechanical response to impact (Halloran et al., 2010). Musculoskeletal models incorporate time-dependent dynamic inputs such as muscle activations, contractions, wrapping, tendon compliances, and joint movement. Furthermore, computational musculoskeletal models are easily scalable to subject-specific anthropometric details and can provide segmental acceleration, velocity, and force information for a specific subject. These features reduce the kinematic errors at the early stage that can propagate throughout a biomechanical analysis. Musculoskeletal models can help us study the cause-and-effect relationship between various muscle and joint physiological properties and head and neck injuries, which is a vital part of this study. Additionally, computational musculoskeletal models are less time-consuming and computationally less expensive than FEM, cadaveric, or dummy tests. Considering all these factors and hypotheses of this study, we employed a musculoskeletal model to investigate the effects of neck strength and impact dynamics on head and neck injury criteria. In our model, we considered three neck strengths: low force capacity (sedentary or untrained), mid force capacity (recreationally active), and high force capacity (Athletic).

To perform a detailed injury analysis, sources of kinematics and kinetics data should be free from bias and cover a broad spectrum of events. The acceleration loading curve on the head has a significant effect on TBI (Post et al., 2014). Musculoskeletal models have been widely used for low-speed human movements like weight lifting, walking, running, etc. Recently, there have been some studies where the musculoskeletal model has been used for moderate severity impacts, such as NFL (Kleiven, 2007; Jin et al., 2017; Mortensen et al., 2018; Kuo et al., 2019; Mortensen et al., 2020) and rugby impacts (Silvestros et al., 2024). Head and neck injuries, because of low and high severity impacts, were not studied much using computational musculoskeletal models. In all those studies, sports impacts were the only source of real-life input data to study the effect of impact dynamics and neck strength on head and neck injury. However, sports data has visual perception and risk compensation phenomenon error (Schmidt et al., 2014). That means players with stronger and larger cervical musculature may be confident that they are more protected from head and neck injuries and may engage in more violent collisions. On the other hand, weaker subjects tend to avoid violent collisions. Furthermore, it was also reported that without visual perception, muscle activation takes 0.027s after an impact, while with visual perception, muscle activation takes 0.127s before the impact (Fanta et al., 2013). It is also reported in the same study that head injury criteria show about 30% decrease with visual perception. For these reasons, the studies and decisions about the effect of impact dynamics and neck strength on head injuries, based only on sports data, may mislead the injury prediction. To fill up these gaps, in our study, we consider three types of impact data: automobile impact test as low severity impact (LSI), NFL as moderate severty impact (MSI), and blast as high severity impact (HSI). Thus, we make our data independent of visual perception error. Details about the severity classification of impact are mentioned in Section 2.3.1.

Another research gap in the literature is that the impacts from all around the head are not widely studied for both head and neck injuries in terms of neck strength. Impact locations influence the head and neck injury metric (Mychasiuk et al., 2016; Yu et al., 2022). Impacts on the frontal or occipital region increase the risk of subdural hematoma, whereas parenchymal contusions are more likely due to the side impacts on the head (Post et al., 2015). In a study, the effects of anterior central, posterior central, lateral central, and posterolateral impacts were analyzed for sports-related concussive and sub-concussive impacts (Mortensen et al., 2020). They found that neck strength has statistical significance in terms of head injury criteria. In another study, they considered cranial anterior, cranial posterior, lateral mid-posterior, mid-anterior, and lateral interior impacts from rugby players (Silvestros et al., 2024). However, they studied only neck injuries; head injuries were not included. Anterior eccentric and posterior eccentric impacts were not studied for both head and neck injuries. However, our hypothesis is that eccentric impact may create higher accelerations and momentum around the head than concentric impacts. To fix this research gap, we considered seven impact locations for all the above-mentioned impact severities and neck strengths: anterior concentric, anterior eccentric, posterior concentric, posterior eccentric, lateral concentric, posterolateral eccentric, and anterolateral eccentric.

To investigate the effect of neck strength and impact dynamics on head and neck injury, a comprehensive study is necessary to examine different neck strengths and consider varying impact severities from all directions to reach a conclusive decision. We hypothesize that eccentric impacts will result in higher GAMBIT and NIC values compared to concentric impacts due to the increased rotational accelerations associated with eccentric loading. In this study, we designed 63 cases guided by these hypotheses (3 impact severities × 3 neck strengths × 7 impact locations). In Section 2, we will provide details about the head-neck dynamics framework, injury criteria, impact dynamics, and simulation workflow. In Section 3, we will present the results, and in Section 4, we will discuss the influence of impact locations, neck strengths, and impact severities on head and neck injury criteria.

## 2 Methods

**FIGURE 1:**
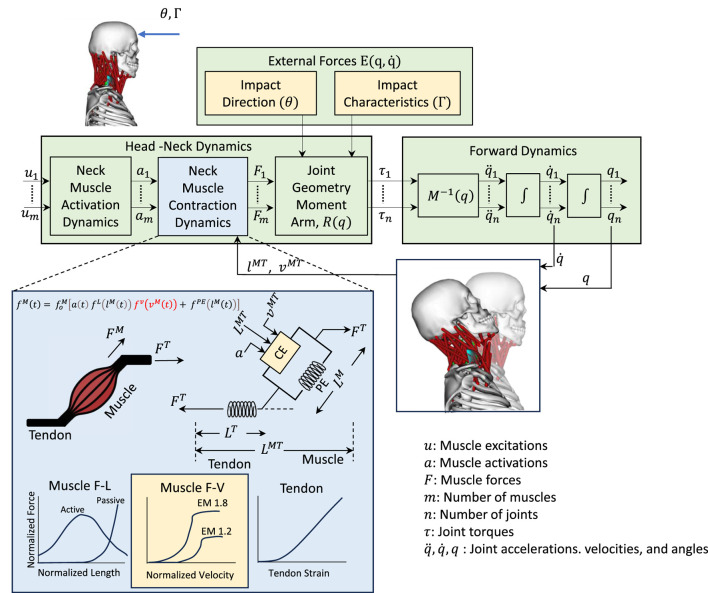
Head-neck dynamics framework uses a muscle-driven simulation to determine the head accelerations. Impact direction ( θ ), impact severiy ( Γ ), and Eccentric multiplier (EM) are considered as design variables of this simulation (yellow color).

The musculoskeletal head-neck model is a muscle-driven model. Neck muscles generate forces based on complex head-neck muscle activation and musculo-tendon dynamics. Under an accelerative environment, all neck muscles collectively generate a muscle-driven head-neck response (head acceleration, muscle strain, and joint reaction load of cervical spine), which is crucial in head and neck injury prediction with high bio-fidelity. The head-neck dynamics framework is provided below in Figure 1.

### 2.1 Head-neck dynamics framework

#### 2.1.1 Head-neck dynamics

The dynamics of the human musculoskeletal system can be formulated with the Euler-Lagrange equations (Zuo et al., 2024) as per Equation 1:

$$M\left(q\right)\ddot{q}=C\left(q,\dot{q}\right)+G\left(q\right)+R\left(q\right)F^{T}\left(u\right)+E\left(q,\dot{q}\right)$$ (1)

Where, q , $\dot{q}$ , and $\ddot{q}$ are the positions, velocities, and accelerations of the joints, $M\left(q\right)$ is the mass distribution matrix which contains masses and inertial properties of the body segments, $C\left(q,\dot{q}\right)$ is the Coriolis and centrifugal force vector which arises when Newton’s laws of motion are applied in reference frames that are fixed to rotating bodies, $G\left(q\right)$ is the gravitational force vector, $R\left(q\right)$ is muscle moment arm matrix, $F^{T}\left(u\right)$ is the tendon force vectors, which is a function of muscle excitations ( u ), $E\left(q,\dot{q}\right)$ is the external forces vector that represents the interactions between the body and environment, including the impact direction ( θ ) and impact severities ( Γ ).

The acceleration of the body in response to muscle forces and other loads can be computed using the equations of motion for the body as per Equation 2.

$$\ddot{q}=M^{-1}\left(q\right)\left\{C\left(q,\dot{q}\right)+G\left(q\right)+R\left(q\right)F^{T}\left(u\right)+E\left(q,\dot{q}\right)\right\}$$ (2)

The process to get the muscle force vectors is shown in Equation 4.

#### 2.1.2 Muscle-force generating parameters

Muscle activation dynamics takes muscle excitation as the input and muscle activation as the output. Muscle excitation $u\left(t\right)$ represents the strength of the excitation signal from the nerve to the muscle, whereas muscle activation $a\left(t\right)$ represents the availability of calcium ions within intracellular space (Uchida and Delp, 2021). The relationship between muscle activation $a\left(t\right)$ and muscle excitation $u\left(t\right)$ can be expressed as a first-order ordinary differential equation, as shown in Equation 3:

$$\dot{a}=\frac{u\left(t\right)-a\left(t\right)}{\tau \left(a,u\right)}$$ (3)

$$where\;\tau \left(a,u\right)=\left\{\begin{array}{l} \tau_{A}\left(0.5+1.5a\left(t\right)\right),if\;u\left(t\right)>a\left(t\right)\\ \frac{\tau_{D}\;}{0.5+1.5a\left(t\right)},otherwise \end{array}\right.$$

$\tau_{A}$ and $\tau_{D}$ represent the activation and deactivation time constants. $\tau_{A}$ is smaller that $\tau_{D}$ . Although the values vary based on age, muscle, composition and other factors, typical values are 10 and 40 ms, respectively (Uchida and Delp, 2021). This relationship shows that the rate of activation slows as the activation level increases due to less amount of calcium release and diffusion. Similarly, the rate of deactivation slows as the muscle activation level decreases. The force-length-velocity relationship of a muscle largely depends on the muscle activation dynamics.

Neck muscle forces can be computed from the activation $a\left(t\right)$ , normalized fiber length $\left(l^{M}\right.$ ), and normalized muscle velocity ( $v^{M}$ ). Muscle force at particular muscle length, velocity and activation is the product of the corresponding values on the force-length and force-velocity curves, as shown in Equation 4:

$$f^{M}\left(t\right)=f_{o}^{M}\left[a\left(t\right)\;f^{L}\left(l^{M}\left(t\right)\right)\;f^{v}\left(v^{M}\left(t\right)\right)+f^{PE}\left(l^{M}\left(t\right)\right)\right]$$ (4)

Where $f^{M}\left(t\right),f_{o}^{M},and\;a$ are fiber force, maximum isometric force, and muscle activation, respectively.

**FIGURE 2:**
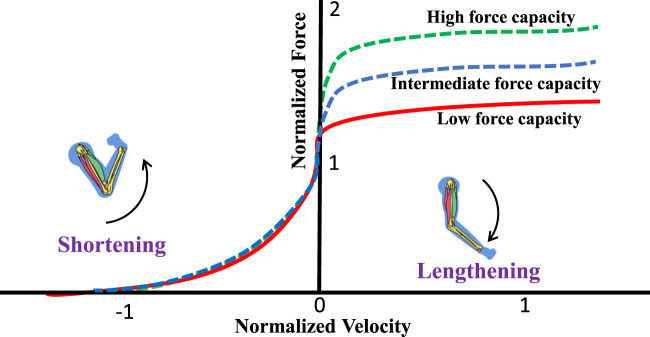
Force-velocity relationship. A higher eccentric multiplier results in higher forces during eccentric (lengthening) contractions. Positive velocity indicates lengthening, and negative velocity indicates shortening.

The total generated neck muscle force is a function of muscle fiber length and muscle velocity. The force-length curve, $f^{L}\left(l^{M}\left(t\right)\right)$ provides the relationship between neck muscle length and generated force at a specific time. The force-velocity curve $f^{v}\left(v^{M}\left(t\right)\right)$ provides the relationship between neck muscle velocity and generated force at a specific time. As our impact severities are classified based on force and duration, we considered neck strength to be a function of the force-velocity curve $f^{v}\left(v^{M}\left(t\right)\right)$ . In musculoskeletal modeling, maximum force output can be expressed as an Eccentric Multiplier (EM) which represents the maximum eccentric contraction velocity, as shown in Figure 2. This parameter ranges between 1.1 and 1.8 and varies from subject to subject.

Based on the eccentric multiplier, we consider three subjects (neck strengths): (i) Low force capacity (Sedentary or untrained): EM = 1.2, (ii) Mid force capacity (Recreationally active): EM = 1.4, and (iii) High force capacity (Athletic): EM 1.8.

#### 2.1.3 Forward dynamics

Forward dynamics takes the musculoskeletal model, initial conditions, muscle excitations, and external forces as the input and provides kinematic data over time, muscle states, and joint torques as the output. It computes muscle force using the Hill-type muscle model (Hill, 1938) based on current states and excitations, solves Equation 2 to get the accelerations, and integrates accelerations over time intervals $\left(\Delta t\right)$ to update velocities ( ${\dot{q}}_{t+1}$ ) and positions $\left(q_{t+1}\right)$ as shown in Equation 5:

$${\dot{q}}_{t+1}={\dot{q}}_{t}+\ddot{q}\;\Delta t$$ (5)

$$q_{t+1}=q_{t}+{\dot{q}}_{t+1}\;\Delta t$$

#### 2.1.4 Joint reaction load

**FIGURE 3:**
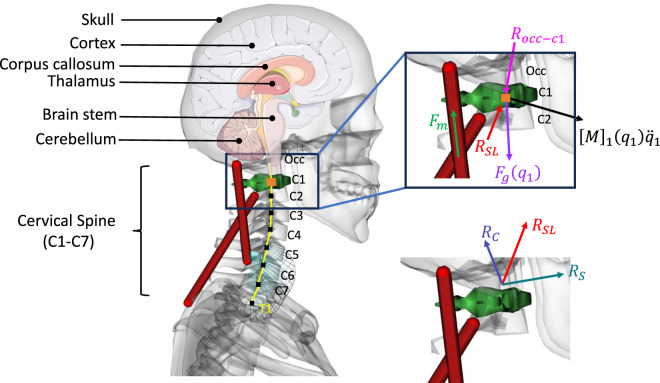
Joint reaction load analysis (simplified C1-C2 joint reaction calculation).

Joint reaction load analysis provides the forces and moments between two consecutive bodies based on the output of forward dynamics. This analysis determines the forces and moments acting on each cervical joint to maintain equilibrium, accounting for external forces, inertial forces, and internal forces, such as force generated from muscles and tendons. It is calculated by solving Newton-Euler equations where all translational and rotational dynamics are presented between those two consecutive bodies. The Newton-Euler Equations for estimating the atlantoaxial (C1-C2) joint reaction load is shown in Equation 6

$$R_{SL}={\left[M\right]}_{1}\left(q_{1}\right){\ddot{q}}_{1}-\sum F_{m}+F_{g}\left(q_{1}\right)+R_{occ-c1}$$ (6)

where ${\left[M\right]}_{1}\left(q_{1}\right)$ is the 6 × 6 mass matrix of cervical spine C1 , $q_{1}$ is the vector of linear and angular displacement of the C1 , ${\ddot{q}}_{1}$ is the vector of the linear and angular accelerations of the C1 , $F_{m}$ are the required muscle forces and moments to follow the given kinematics, $F_{g}\left(q_{1}\right)$ is the gravitational loading, $R_{occ-c1}$ is the atlanto-occipital (O-C1) joint reaction force and moment, which can be found from the similar step for upper bodies, and $R_{SL}$ is the atlantoaxial (C1-C2) joint reaction force and moment. $R_{c}$ and $R_{s}$ are the compressive and shear forces on the atlantoaxial (C1-C2) joint in Figure 3.

### 2.2 Integration of head, neck, and muscle injury criteria with the framework

#### 2.2.1 Head injury criteria

Acceleration is measured in multiples of the acceleration of gravity (g), and time is measured in seconds. Head Injury Criteria (HIC) is proposed by the US National Highway Traffic Safety Administration (NHTSA), as shown in Equation 7

$$HIC=\max{\left[\frac{1}{t_{2}-t_{1}}\;\int_{t_{1}}^{t_{2}}a\left(t\right)dt\;\right]}^{2.5}\left(t_{2}-t_{1}\right)$$ (7)

Where, $t_{1}$ and $t_{2}$ represent two arbitrary time points during the acceleration pulse. Acceleration is measured in gravitational force ( g ), and time is measured in seconds. $HIC_{36}$ means that HIC is measured for each 36 ms apart ( $t_{2}$ and $t_{1}$ is less than 36 ms apart). The HIC model is developed based on animal and cadaver experiments. Suggested human tolerance for 50th percentile males are: $HIC_{36}<1000\text{ and}$ $HIC_{15}<700$ . According to a study, $HIC_{36}<1000$ represents 18% probability of a severe head injury, 55% probability of serious injury, and 90% probability of a moderate head injury to the average adult (Mackay, 2007). In another study, concussions were found at $HIC_{15}=250$ in most athletes (Viano, 2005; Viano and Pellman, 2005).

However, rotational acceleration is responsible for shear stress that damages brain tissue. HIC does not consider rotational acceleration of the head in its injury evaluation. The Generalized Acceleration model Brain Injury Threshold (GAMBIT) model was developed by Newman (Newman, 1986) by combining translational and rotational components of head acceleration as shown in Equation 8.

$$GAMBIT={\left[{\left(\frac{a\left(t\right)}{a_{c}}\right)}^{n}+{\left(\frac{\ddot{\phi }\left(t\right)}{\ddot{\phi_{c}}}\right)}^{m}\right]}^{\frac{1}{k}}$$ (8)

Where $a\left(t\right)$ and $\ddot{\phi }\left(t\right)$ denote the translational and rotational acceleration, respectively. $a_{c}$ and ${\ddot{\phi }}_{c}$ represent critical tolerance levels of those accelerations. It is reported that GAMBIT is one of the strongest predictors of human mTBI (Hernandez et al., 2015). Newman (1986) proposed 250 g and 10,000 $rad/s^{2}$ as their critical thresholds. n,m, and k are constants (Zhan et al., 2021). A GAMBIT value of G = 1 represents a 50% probability of serious brain injury (Feist et al., 2009). Recent studies propose 80–100 g for linear accelerations, and 5,000-6,500 $rad/s^{2}$ for 50% risk of mTBI (Funk et al., 2007; Rowson et al., 2012). However, to the best of authors’ knowledge, there is no contemporary studies that tried to connect the revised threshold based GAMBIT rating with Abbreviated Injury Scale (AIS) or probability of TBI risk. For this reason, in this study, we used the legacy thresholds 250 g and 10,000 $rad/s^{2}$ for linear and rotational accelerations, respectively, as per the literature data we found (Chinn et al., 2001; Fernandes and Sousa, 2015; Schmitt et al., 2019). In this study, we assess the effect of impact locations. The eccentric impacts create significant rotational accelerations. As HIC does not consider rotational acceleration, we used GAMBIT to assess the head injury risk. However, as the GAMBIT threshold varies from study to study, we used HIC values to validate our data in comparison with the literature data.

Margulies and Thibault (1992) reported that the threshold value of shear strain has been reported to be about 5%–10% in case of DAI. It was observed that 50 percent concussion probability is observed within the corpus callosum at 21 percent strain (Kleiven, 2007). Deck and willinager proposed a 31% for maxmimum principal strain threshold and a 25% shear strain to indicate the onset of axonal damage. Rika carlsen adopted tissue level threshold 18% as the axonal strain injury tolerance. Bain et al. considered 18% strain as for the onset of electrophysiological impairment and 21% as morphological damage of axon. Kimapra et al. found out that at 63 g and 4,267 rad/s^2 accelerations, the brain tissue strain is about 29% for 25% mTBI. However, the macro level paramters and tissue level paraterms interactions requuiires further researche to devleop the bridge.

#### 2.2.2 Neck injury criteria

The existing understanding of neck injury criteria is based on load combinations of axial force and flexion-extension moments that are measured with ATDs. Studies have shown that a compressive force of 1750 N–4800 N can cause cervical disc injury (Myers and Winkelstein, 1995; Whyte et al., 2020). One of the most utilized neck injury criteria is $N_{ij}$ (Prasad and Daniel, 1984) which is adopted by the US National Highway Traffic Safety Administration (NHTSA) for assessing automobile injuries. $N_{ij}$ is based on linear combination of axial force and bending moment. However, the neck injury criteria are based on force and moments, and the injuries are completely ignored due to coronal plane loads that cause lateral bending. As external impacts for this study are horizontal and not compressive forces, we considered relative velocity and acceleration based neck injury criteria (NIC) (Boström and Kullgren, 2007). The Neck Injury Criteria (NIC) is shown in Equation 9:

$$NIC\;\left(t\right)=H\;*\;a_{rel}\left(t\right)+v_{rel}{\left(t\right)}^{2}$$ (9)

$a_{rel}\left(t\right),v_{rel}\left(t\right)$ = Relative acceleration and velocity between the first thoracic vertebra (T1) and first cervical spine (C1), H is the neck length (Boström and Kullgren, 2007). Injury prediction is done considering the pressure gradient caused by a sudden change of the fluid flow inside the fluid compartment of the cervical spine. Bohman and Haland (2000) suggested NIC value $15\;m^{2}/s^{2}$ for AIS1 (minor injury). $N_{ij}$ is based on compressive force and moments on cervical spine and considers only flexion and extension related injury. Therefore, we considered NIC as we have lateral impacts, and it creates rotational movement along with flexion and extension.

#### 2.2.3 Muscle-tendon strain

Muscle-tendon strain is expressed as a percentage increase of its current length compared to the length when it is at rest and developing no force. The resting length is called the slack length. The strain of muscle-tendon at any given instant can be expressed in Equation 10.

$$\epsilon^{MT}=\frac{l^{MT}-l_{s}^{MT}}{l_{s}^{MT}}$$ (10)

Here, $l^{MT}$ is muscle-tendon current length, and $l_{s}^{MT}$ is muscle-tendon slack length. In this study, we considered three hyoid muscles and three superficial multifidus muscles strain to assess the muscle injury probability. Three hyoid muscles: sternohyoid, sternothyroid, and omohyoid. The superficial multifidus muscles are superficial multifidus (C5/6-C2), superficial multifidus (C6/7-C2), and superficial multifidus (T1-C4). For more than 10% strain, tendon begins to experience mechanical failure, and there is a high risk of injury (Uchida and Delp, 2021).

### 2.3 Impact parameters

#### 2.3.1 Impact severities ( Γ )

We adopted the classification of impact severity from Beeman et al. (2012). Based on impact durations and force, we classified external forces into three categories: (i) Low-severity impact (LSI), $\Gamma_{1}$ : impact force <1500 N and impact durations >30ms , (ii) Medium severity impact (MSI), $\Gamma_{2}$ : (impact force 1500−3000 N and impact durations <30ms ), (iii) High severity impact (HSI), $\Gamma_{3}$ : impact force >3500 N and impact durations <5ms . Average low velocity (<35 mph) automobile barrier impact tests conducted by the National Highway Traffic Safety Administration (NHTSA) were considered for LSI (National Highway Traffic Safety Administration (NHTSA)), as the impact duration is longer and the change of velocity and impact force are low during the test. American football head impacts data were used for the MSI as the impact forces are higher, the impact duration is shorter, and severity is moderate (Mortensen et al., 2020). Blast data were used as HSI as the force is very high, the impact duration is very short, and the severity is highest (Kulkarni et al., 2014). However, there is no standard classification of external forces. The impact severity is classified to test the hypotheses of this study.

#### 2.3.2 Impact locations ( θ )

The selection of impact locations was driven by both clinical injury prevalence and biomechanical considerations. These locations encompass a comprehensive range of impact directions observed in high-energy events such as automotive crashes, sports collisions, and blasts (Pellman et al., 2003; Post et al., 2015; National Highway Traffic Safety Administration, 2020). Post et al. (2015) reported that subdural hematoma occurred from impacts to the frontal and occipital regions, whereas impacts to the sides of the head produced parenchymal contusions than the impacts to the front and occipital regions of the head. Pellman et al. (2003) indicated that 78% of NFL impacts happen from the frontal left and right sides.

**FIGURE 4:**
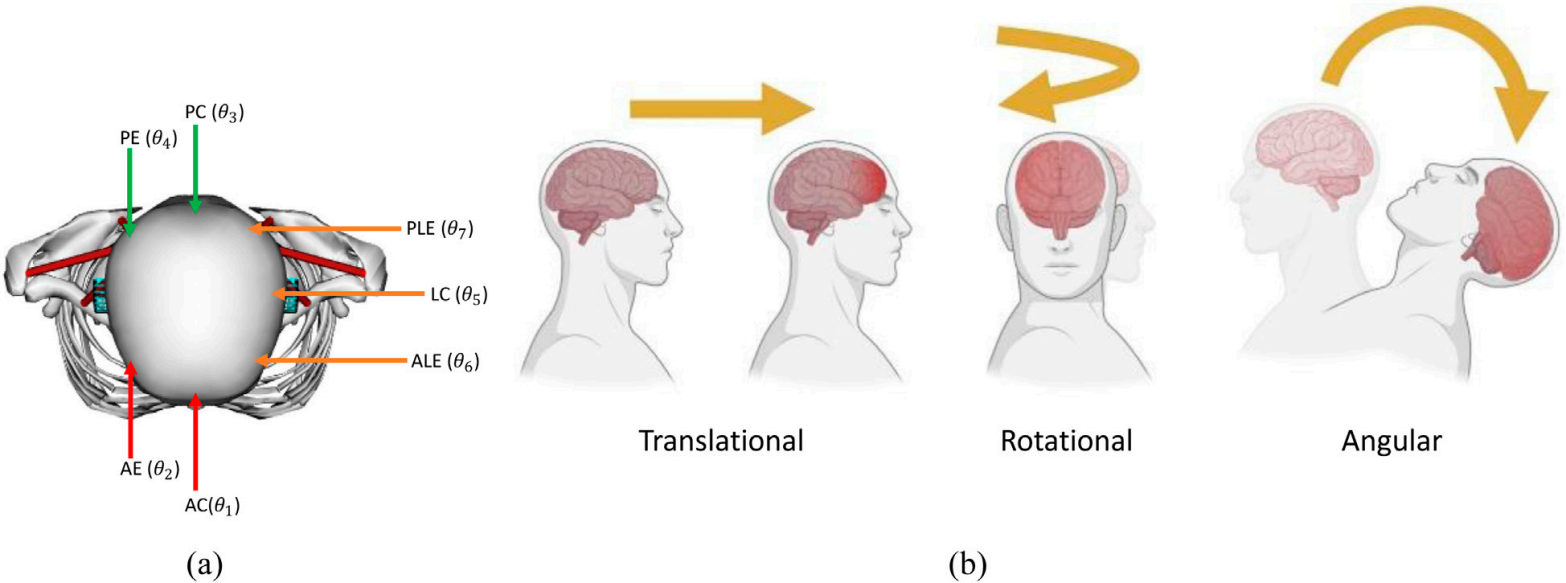
**(a)** Impact locations (AC = Anterior Concentric, AE = Anterior Eccentric, PC = Posterior Concentric, PE = Posterior Eccentric, LC = Lateral Concentric, PLE = Posterolateral Eccentric, ALE = Anterolateral Eccentric), **(b)** types of head acceleration.

We categorized the location of head contact into seven impact locations ( θ ). Two anterior impacts, two posterior impacts, and three lateral impacts. We chose two impact locations for frontal and rear impacts, as head and neck anatomy are symmetric with respect to the sagittal plane. On the other hand, we choose three lateral impacts as head anatomical properties and neck muscles are asymmetric with respect to coronal plane. Figure 4a shows the impact locations. The two anterior impacts ( $\theta_{1},\theta_{2}$ ) are anterior central (AC) and anterior eccentric (AE). The two posterior impacts ( $\theta_{3},\theta_{4}$ ) are posterior central (PC) and posterior eccentric (PE). The lateral impacts ( $\theta_{5},\theta_{6}$ , $\theta_{7}$ ) are lateral central (LC), anterolateral eccentric (ALE), and posterolateral eccentric (PLE). Seven impact locations cover key anatomical planes. We choose ALE and PLE lateral eccentric directions as it is reported that 57% of the NFL impacts involve these regions (Pellman et al., 2003).

There are three different types of acceleration that affect the skull and the head: translational, rotational, and angular acceleration, as shown in Figure 4b. For the translational type, also known as linear, the center of gravity of the head will move in a straight line without rotation, and this usually results in focal injury, while in rotational acceleration, there is no movement of the center, but the head will rotate around it, resulting in diffuse shear strain. Lastly, the angular type is a combination of both rotational and linear properties, and the center of gravity moves in an angular manner. The angular type is the most common type and is responsible for the bulk of the TBI cases.

### 2.4 Simulation workflow

#### 2.4.1 Data collection

We collected three datasets to analyze the relationship of neck strength, impact locations, and impact severities on head and neck injury parameters. For the low-severity impacts (LSI), we collected an automotive crash test from the NHTSA database. The test number was 11,149 (National Highway Traffic Safety Administration, 2020). A frontal rigid barrier impact test was conducted on a 2020 Toyota Highlander SUV. The impact velocity of the vehicle was 56.35 km/h (35 mph). A 50th percentile male Hybrid III ATD was placed in the driver sitting position. The impact duration was about 75 ms, and the impact force was 1210N. For the moderate-severity impacts (MSI), we used extrapolated American Football data (Mortensen et al., 2018). The impact duration was 13 ms, and the maximum impact force was 1510N. For the high-severity impacts (HSI), we used simulated blast data (Kulkarni et al., 2014). The impact duration was 5 ms and the impact force was 3455N. The exact force and time duration data were also used to validate the model.

#### 2.4.2 Musculoskeletal model simulation

A modified head-neck musculoskeletal model was employed in OpenSim software (Delp et al., 2007; Seth et al., 2018). The model was verified using 10 subjects’ data for moderate severity impacts. The head neck model is based on Mortensen (Mortensen et al., 2018) and Vasavada’s model (Vasavada et al., 1998). The model has a total of 72 Millard muscle actuators (Millard et al., 2013; Kuo et al., 2019). Neck muscles such as multifidus and Semispinalis Cervicis are short and highly pennated. The Millard muscles better capture muscle-specific behaviors, such as tendon compliance and fiber pennation angles. Moreover, the Millard model has detailed non-linear tendon dynamics. Therefore, it reflects neck muscle activation dynamics better than traditional hill-type muscle. The musculoskeletal model incorporates soft-tissue damping and rate-dependent ligaments. It also includes passive cervical spine ligaments and viscoelastic properties of muscles. These passive components stabilize the cervical spine during dynamic loading, ensuring biofidelic GAMBIT and NIC predictions for lateral and flexion-extension impacts. These properties are crucial for accurately simulating energy dissipation during rapid stretching in impacts. The details about the model can be found in (Kuo et al., 2018; Mortensen et al., 2018; Kuo et al., 2019; Mortensen et al., 2020).

**FIGURE 5:**
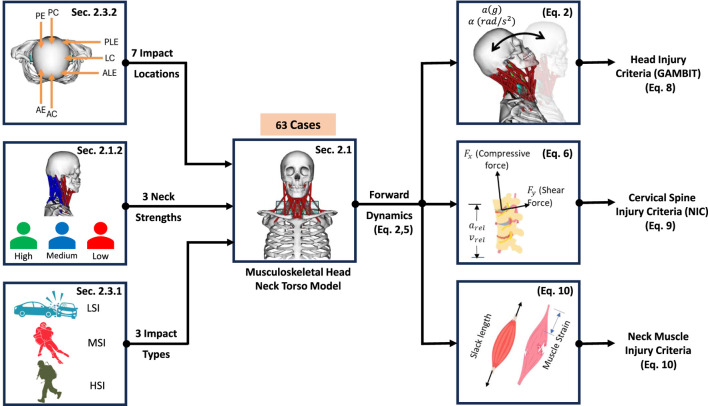
Musculoskeletal model simulation workflow for 63 cases.

Muscle activation data for neck stiffness were collected from the literature (Kuo et al., 2018; Kuo et al., 2019). Three neck strengths, three impact severities, and seven impact locations, a total of 63 cases, were performed. A MATLAB script was used to run these 63 cases in OpenSim forward dynamics simulations. We used OpenSim’s adaptive time step size for the forward dynamics. The maximum step size was set to 1, and the minimum step size was set to 1 × 10^−7^. The integrator tolerance was set to 1 × 10^−5^. During the forward dynamics, the integrator dynamically chooses a step size between these bounds to satisfy the integrator error tolerance. For slower and steadier motion, it will increase the step size to save computation time. For highly dynamic simulations, the step size shrinks to capture fast-changing dynamics. The workflow diagram is shown in Figure 5.

#### 2.4.3 Statistical analyses

Statistical analyses were performed using IBM SPSS Statistics version 29.0 (IBM Corp., Armonk, NY, USA). Initially, normality of the data was assessed with the Shapiro-Wilk test. Parametric one-way analysis of variance (ANOVA) was employed for normally distributed data, while the nonparametric Kruskal–Wallis test was utilized for datasets deviating from normality. For pairwise comparisons, the Mann-Whitney U test was conducted.

A *post hoc* power analysis for GAMBIT, conducted in SPSS using an effect size of f = 10.46 for the observed means (LSI: 0.6179, MSI: 0.8934, HSI: 0.9542; SDs: 0.0252, 0.0735, 0.0288; Table 3), indicates that n = 3 per neck strength category ( N = 27 total) achieves a power of 1.000 for one-way ANOVA ( α = 0.05, two-tailed). For a 20% difference in GAMBIT ( f = 1.53), power is 1.000, confirming robustness for significant findings reported in Section 4.3.

## 3 Results

### 3.1 Head injury risk analysis based on linear and rotational accelerations

**FIGURE 6:**
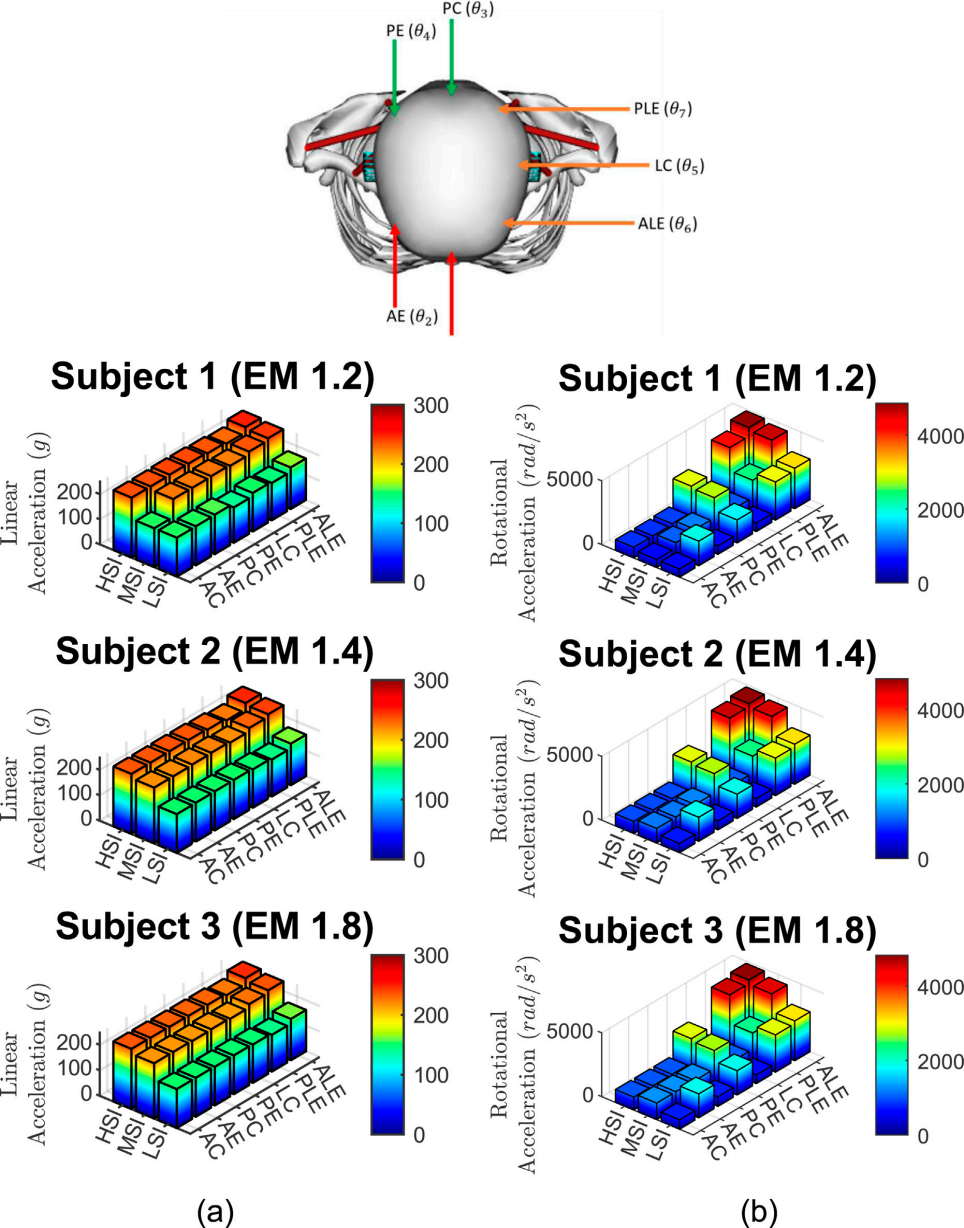
Accelerations from 63 cases, **(a)** Linear accelerations and **(b)** rotational accelerations for three subjects, three impact severities, and seven impact locations.

The linear accelerations are presented in Figure 6a. The rotational accelerations are presented in Figure 6b. The linear and rotational accelerations of the head are calculated at its center of mass.

### 3.2 Head injury risk analysis based on GAMBIT

**FIGURE 7:**
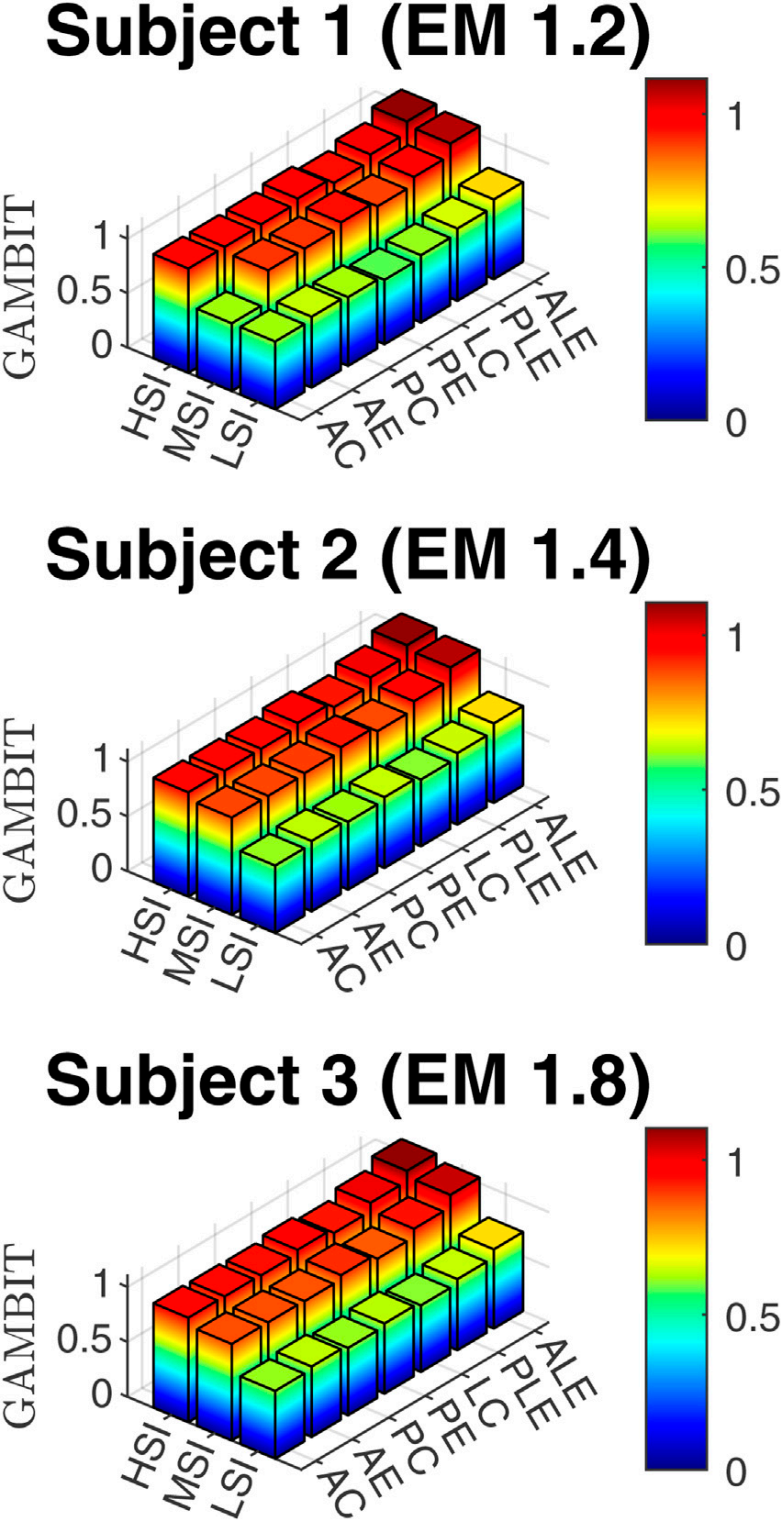
GAMBIT for three subjects, three impact severities, and seven impact locations.

The GAMBIT results, based on Equation 7 are presented in Figure 7.

### 3.3 Neck injury risk assessment based on cervical spine force and moment

**FIGURE 8:**
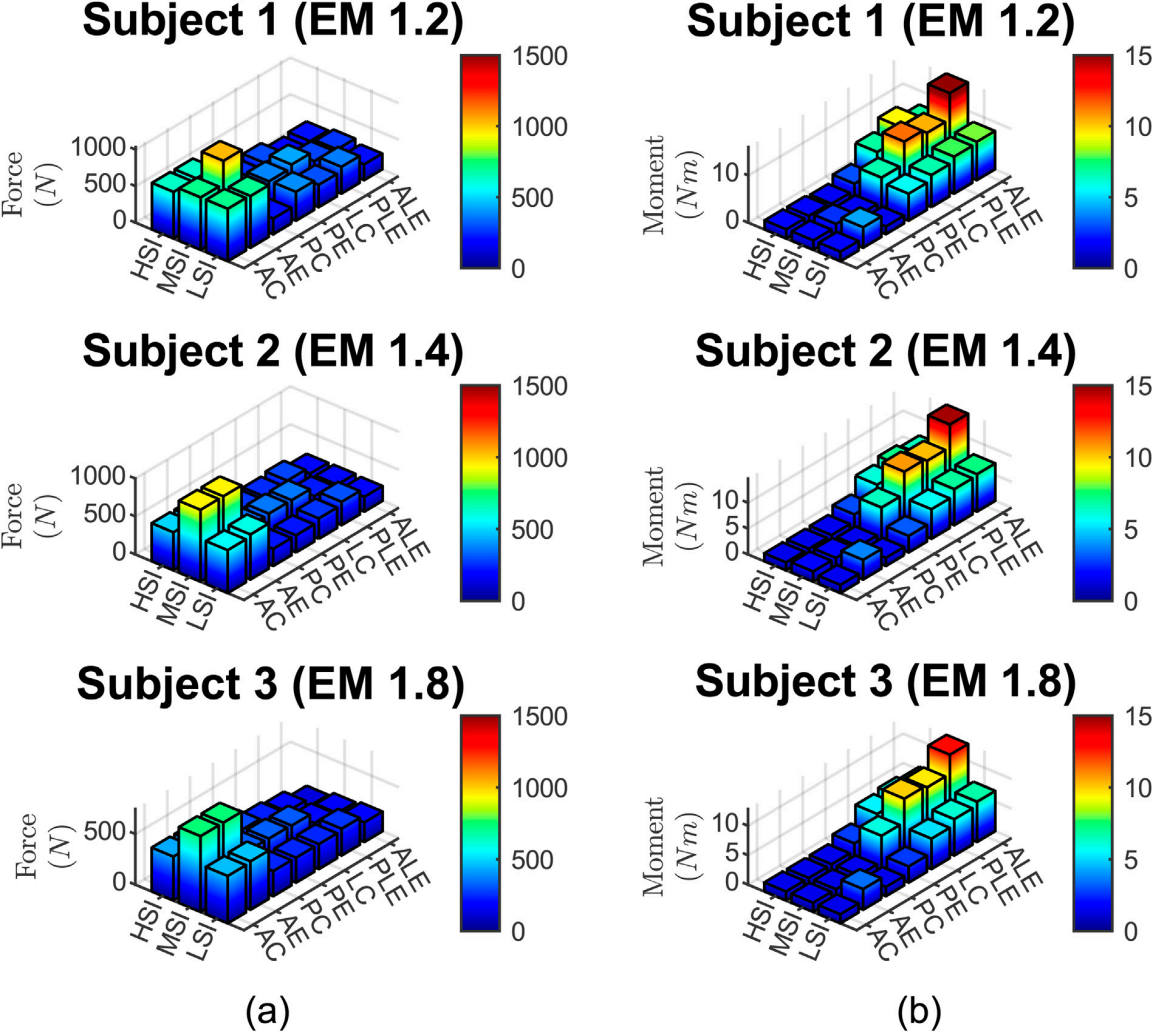
**(a)** neck force, **(b)** neck moment for three subjects, three impact severities, and seven impact locations.

The calculated neck force and moment data are presented in Figure 8.

### 3.4 Neck injury assessment based on NIC

**FIGURE 9:**
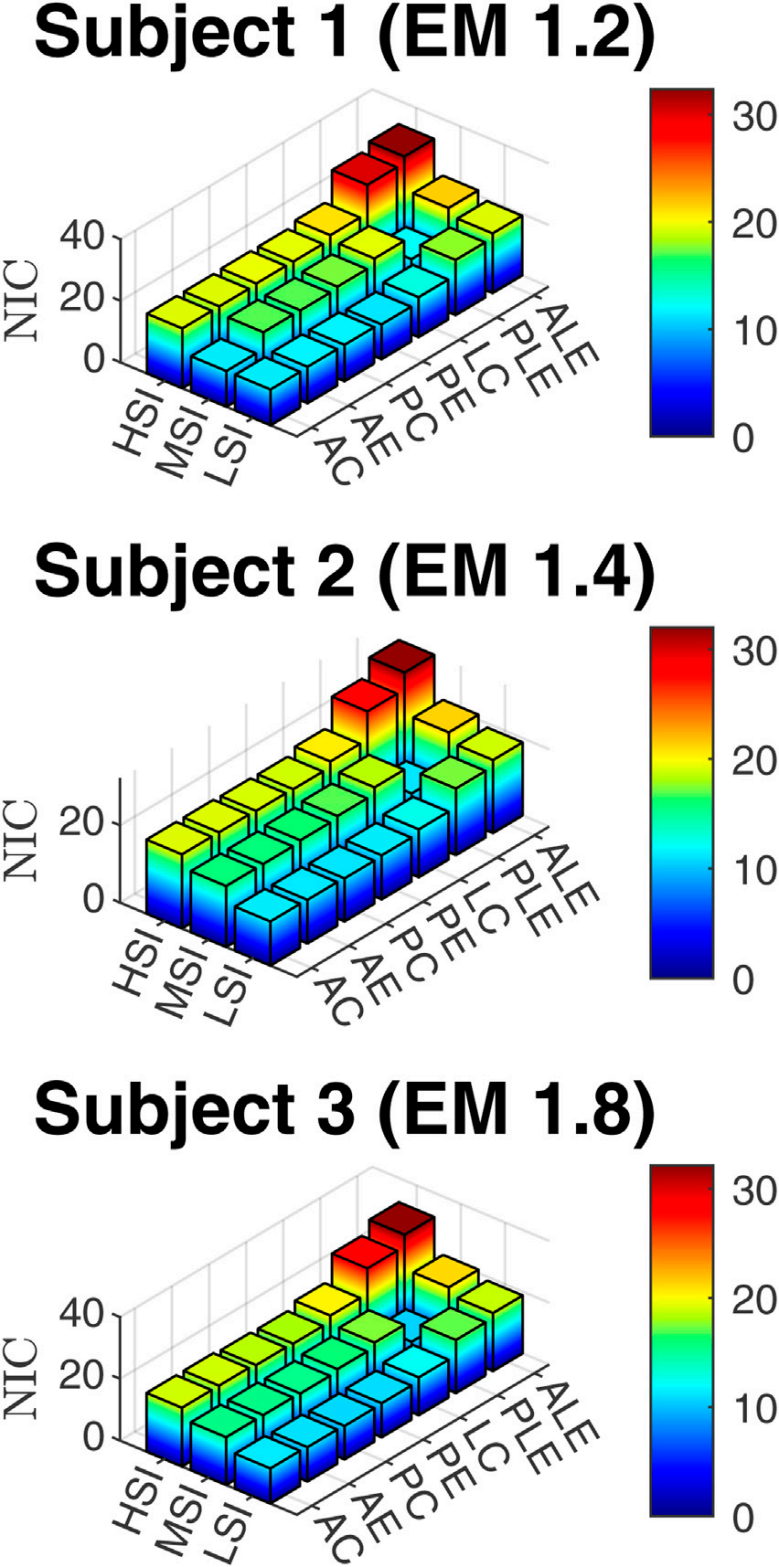
NIC for three subjects, three impact severities, and seven impact locations.

The NIC data, based on Equation 8 are presented in Figure 9.

### 3.5 Muscle strain injury

**FIGURE 10:**
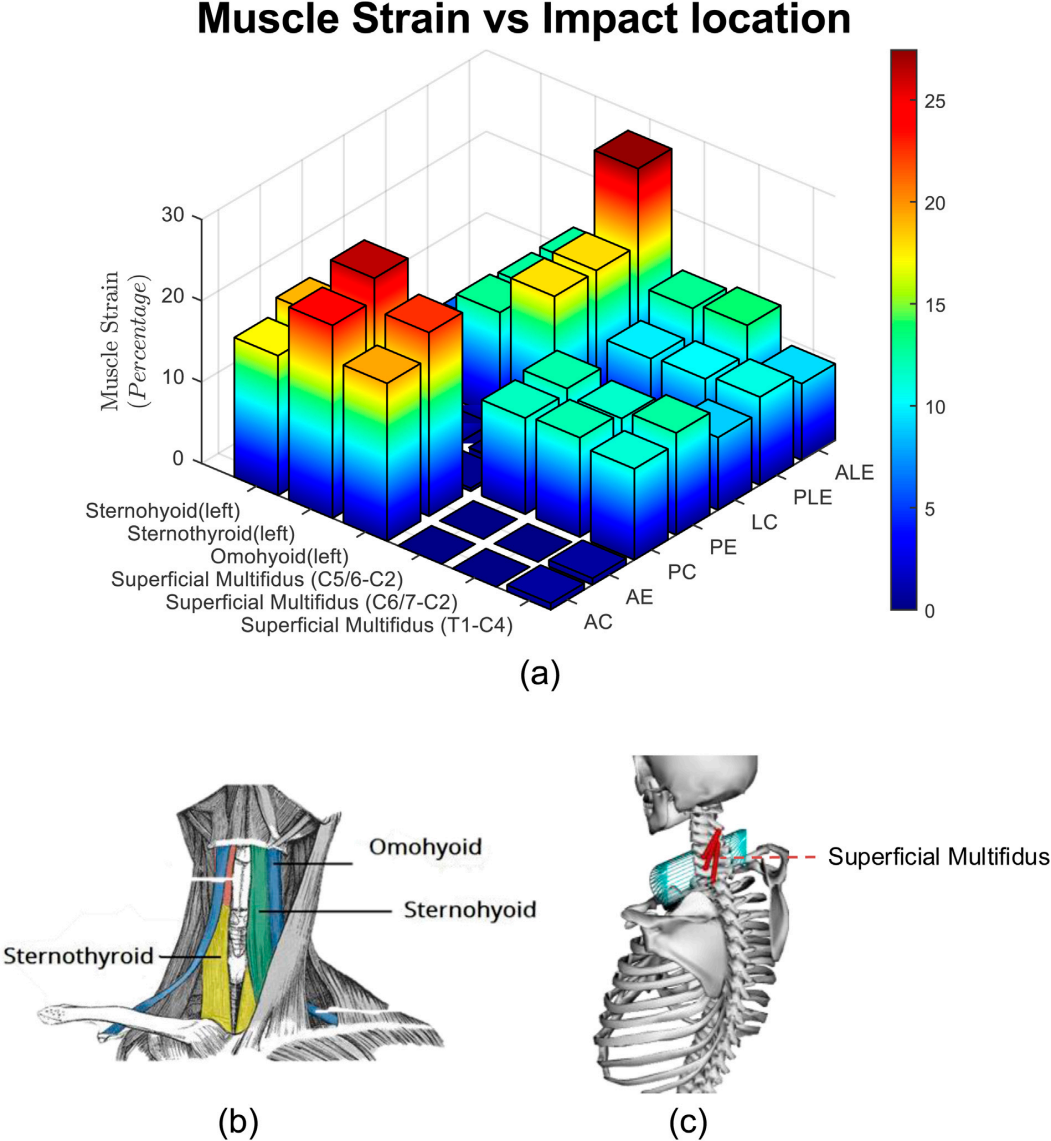
**(a)** Muscle Strain for three subjects, three impact severities, and seven impact locations. **(b)** Hyoid muscles, **(c)** superficial multifidus muscles.

The strains for six different muscles, that take part in resistive action of the neck are presented in Figure 10.

## 4 Discussion

In this study, we developed a computational musculoskeletal head-neck model using OpenSim software to investigate the effects of impact location, neck strength, and impact severity on head and neck injury parameters. We designed a total of 63 simulation cases, varying across seven impact locations, three neck strength levels, and three impact severities.

### 4.1 Effect of impact location

**FIGURE 11:**
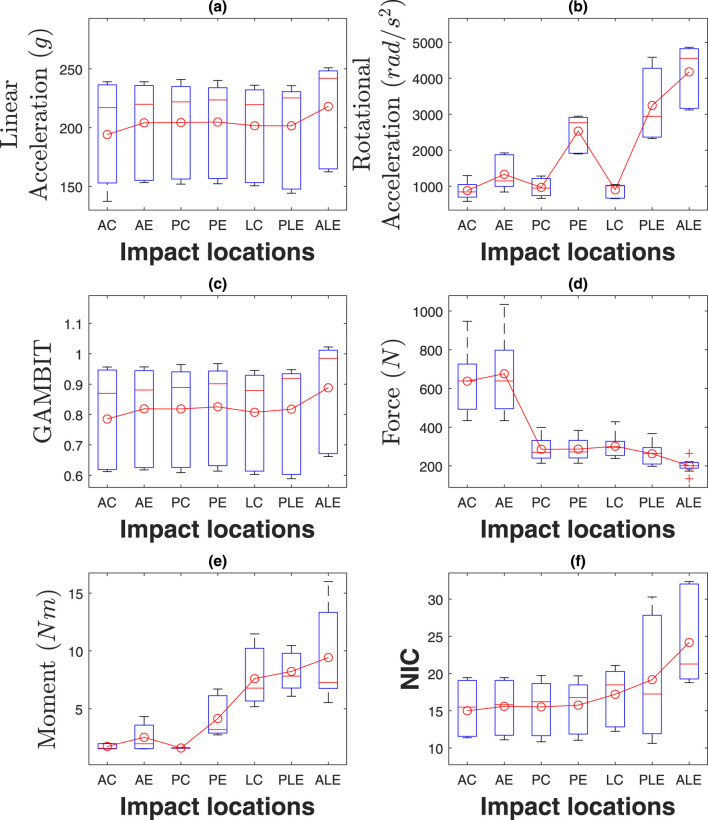
Effect of impact locations on **(a)** linear acceleration, **(b)** rotational acceleration, **(c)** GAMBIT, **(d)** neck force, **(e)** neck moment, and **(f)** NIC.

The box plots in Figure 11 illustrate the linear and rotational accelerations, GAMBIT, neck force, neck moment, and NIC across various impact locations, with data taken from three subjects. The boxes represent the interquartile range (IQR), the red horizontal lines indicate the medians, and the red circles show the mean values, while the whiskers display the minimum and maximum values within the data range. Table 1 shows the mean value of each data for different locations. It can be seen from 9 (a) that linear accelerations do not vary much based on impact directions and locations and stay within 194 g–218 g. Eccentric impacts create higher linear acceleration than central impacts however, the increment is less than 10%. The lateral impacts generate about 10% more linear accelerations than anterior and posterior impacts. The hyoid muscles are highly stretched (>10%) during frontal and ALE impacts, whereas the multifidus muscles were highly stretched during posterior and lateral impacts.

**TABLE 1:** Effect of impact locations.

Parameter	Anterior central	Anterior eccentric	Posterior central	Posterior eccentric	Lateral central	Posterolateral eccentric	Anterolateral eccentric
a ( g )	194	204	204	204	201	201	218
α ( $rad/s^{2}$ )	879	1,330	966	2,531	901	3,244	4176
GAMBIT	0.79	0.82	0.82	0.83	0.81	0.82	0.89
$F_{neck}$ ( N )	638	676	286	287	302	263	201
$M_{neck}$ ( Nm )	1.75	2.54	1.61	4.16	7.61	8.23	9.43
NIC	14.9	15.59	15.52	15.75	17.21	19.18	24.17

To quantify the effect of impact location on injury parameters, we conducted Kruskal–Wallis tests due to normality violations (Shapiro-Wilk p < 0.05 for most variables). Significant main effects were found for NIC ( $\chi^{2}\left(6\right)$ = 16.650, p = 0.011), rotational acceleration ( $\chi^{2}\left(6\right)$ = 49.357, p < 0.001), neck force ( $\chi^{2}\left(6\right)$ = 45.771, p < 0.001), and neck moment ( $\chi^{2}\left(6\right)$ = 48.720, p < 0.001), but not for GAMBIT ( p = 0.266) or linear acceleration ( p = 0.289). Pairwise Mann-Whitney U tests (Bonferroni-corrected, p < 0.025) comparing anterolateral eccentric (ALE) to anterior central (AC) and anterior eccentric (AE) impacts confirmed significant differences for NIC (ALE: 21.28 $m^{2}/s^{2}$ vs. AC: 15.64 $m^{2}/s^{2}$ , p = 0.004; vs. AE: 15.80 $m^{2}/s^{2}$ , p = 0.002), rotational acceleration (ALE: 4,554.66 $rad/s^{2}$ vs. AC: 893.20 $rad/s^{2}$ , p < 0.001; vs. AE: 1,148.54 $rad/s^{2}$ , p < 0.001), neck force (ALE: 201.10 N vs. AC: 602.64 N, p < 0.001; vs. AE: 638.88 N, p < 0.001), and neck moment (ALE: 7.27 Nm vs. AC: 1.57 Nm, p < 0.001; vs. AE: 1.98 Nm, p < 0.001). These results confirm that ALE impacts produce significantly higher rotational acceleration and neck moment, supporting the claim of increased injury risk for anterolateral impacts. No significant differences were found for GAMBIT (ALE: 0.98 vs. AC: 0.88, p = 0.046; vs. AE: 0.88, p = 0.050) or linear acceleration (ALE: 241.98 $m^{2}/s^{2}$ vs. AC: 218.52 $m^{2}/s^{2}$ , p = 0.059; vs. AE: 219.92 $m^{2}/s^{2}$ , p = 0.063). The non-significant results for GAMBIT suggest that GAMBIT is more sensitive to the magnitude of acceleration (severity) than its directional application (location) (1.44x and 1.54x higher for MSI and HSI vs. LSI, as mentioned in Section 4.3). Also, it indicates that head injuries are more sensitive to rotational accelerations than linear accelerattions which aligns with previous studies (Wright, 2012; Wright et al., 2013). Anterolateral eccentric (ALE) impacts exhibited greater consistency in neck force values, suggesting a stable biomechanical response that may contribute to their classification as the most risky impact location compared to other locations.

The rotational accelerations in Figure 11b vary significantly based on impact directions and locations. The eccentric impacts generated about 1.51, 2.61, and 4.11 times more rotational acceleration than central impacts, for the anterior, posterior, and lateral sides, respectively. Posterior and lateral impacts create 1.58 and 2.51 times more rotational acceleration than anterior impacts, respectively. The maximum average rotation acceleration is for anterolateral eccentric impact (4,176 $rad/s^{2}$ ) which is 4.75 times more than the average anterior central impact (879 $rad/s^{2}$ ). The rotational acceleration of 4,267 $rad/s^{2}$ has 25% chance of mTBI (King, 2018; Zaman et al., 2024). Translational or linear accelerations cause the focal injuries to the brain, such as contusion and hematoma, whereas rotational acceleration causes diffuse injuries, such as DAI and subdural hematoma. Rotational accelerations create multidirectional strain fields that primarily affect the white matter structures such as axons. Margulies and Thibault (Margulies and Thibault, 1992) reported that the threshold for shear strain associated with diffuse axonal injury (DAI) ranges from 5% to 10%. Kleiven (2007) mentioned that a 50% concussion probability occurs within the corpus callosum at a 21% strain level. Deck and Willinger (2008) proposed a maximum principal strain threshold of 31% and a shear strain threshold of 25% to indicate the onset of axonal damage. Write et al. (2013) adopted an axonal strain injury tolerance at 18%, while Bain and Meaney (2000) identified 18% strain as the threshold for electrophysiological impairment and 21% for morphological axonal damage. Kimpara and Iwamoto (2012) determined that brain tissue strain reaches approximately 29% under accelerations of 63g and 4,267 rad/s^2^, corresponding to a 25% mTBI risk. However, further research is needed to clarify the interplay between macro-level parameters and tissue-level metrics to establish a comprehensive link between these scales.

The GAMBIT values in Figure 11c do not vary very much with the impact directions and locations similar to linear accelerations. It was within 0.79–0.83 for all impact locations except for anterolateral eccentric, which was 0.89. In summary, impact locations have a significant effect on rotational acceleration, and anterolateral impact is the riskiest impact location for both head and neck.

The average neck forces for the anterior impacts were about 2.5 times higher than for posterior and lateral impacts. The maximum average neck force was for the anterior eccentric impacts (676N). Neck moments in Figure 11e vary significantly based on impact locations and directions. The highest average neck moment was for anterolateral eccentric (9.43Nm), which was 5.85 times more than the posterior central neck moment (1.61Nm). Also, eccentric impacts generate about 157% more neck moments than central impacts. NIC values in Figure 11f were within 15–24 $m^{2}/s^{2}$ . The lateral impact values were higher than the anterior and posterior impacts.

### 4.2 Effect of neck strength

**FIGURE 12:**
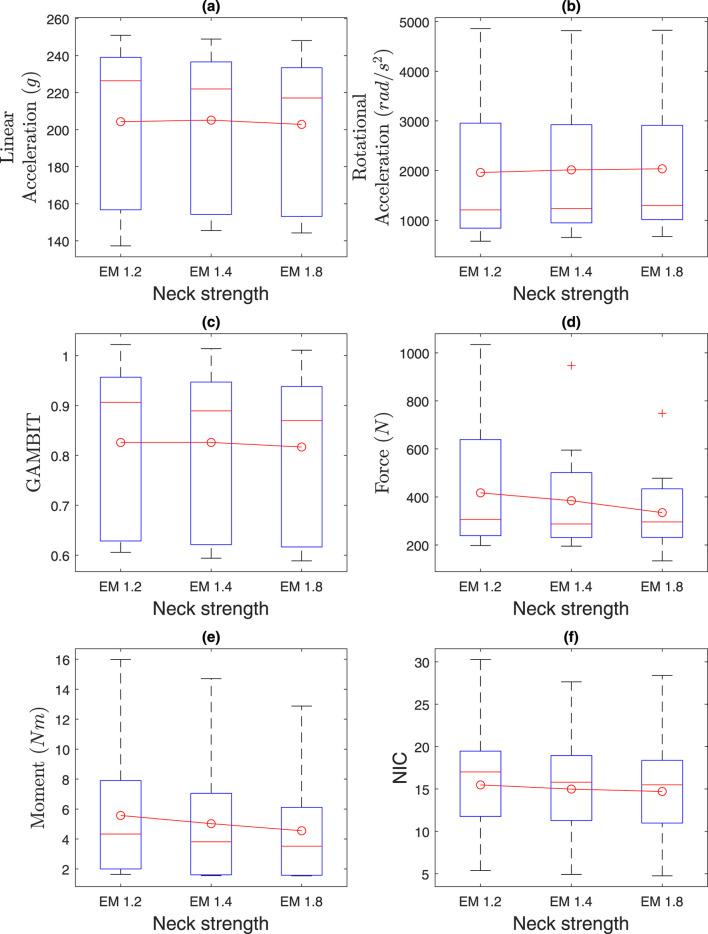
Effect of neck strength on **(a)** linear acceleration, **(b)** rotational acceleration, **(c)** GAMBIT, **(d)** neck force, **(e)** neck moment, and **(f)** NIC.

The effects of neck strength are presented in Figure 12 and Table 2. Average linear accelerations in Figure 12a do not vary much with neck muscle strength and were within 202 g to 205 g . The average rotational accelerations in Figure 12b also do not vary much and stay between 1960 $rad/s^{2}$ to 2037 $rad/s^{2}$ . The average GAMBIT in Figure 12c ranges from 0.81-0.83.

**TABLE 2:** Effect of neck strength.

Parameter	Low force capacity (EM 1.2)	Mid-force capacity (EM 1.4)	High force capacity (EM 1.8)
a	204	205	202
α	1962	2014	2036
GAMBIT	0.82	0.82	0.81
$F_{neck}$	417	385	335
$M_{neck}$	5.56	5.02	4.54
NIC	15.4	14.98	14.7

Kruskal–Wallis tests showed no significant effects of neck strength (EM 1.2, 1.4, 1.8) on any injury parameters ( p > 0.184 for linear acceleration, rotational acceleration, GAMBIT, neck force, neck moment, and NIC), consistent with the minimal variation ( < 10 % ) in medians and means across neck strength levels.

The average neck force in Figure 12d ranges from 334N to 417N. The average neck moment in Figure 12e ranges from 4.5Nm to 5.6Nm. The average NIC value ranges from 14.7 to 15.5. In summary, the average head and neck injury parameters do not vary by more than 10% based on neck strength, which agrees with the literature (Mortensen, Vasavada et al., and statistical analysis.

### 4.3 Effect of impact severities

**FIGURE 13:**
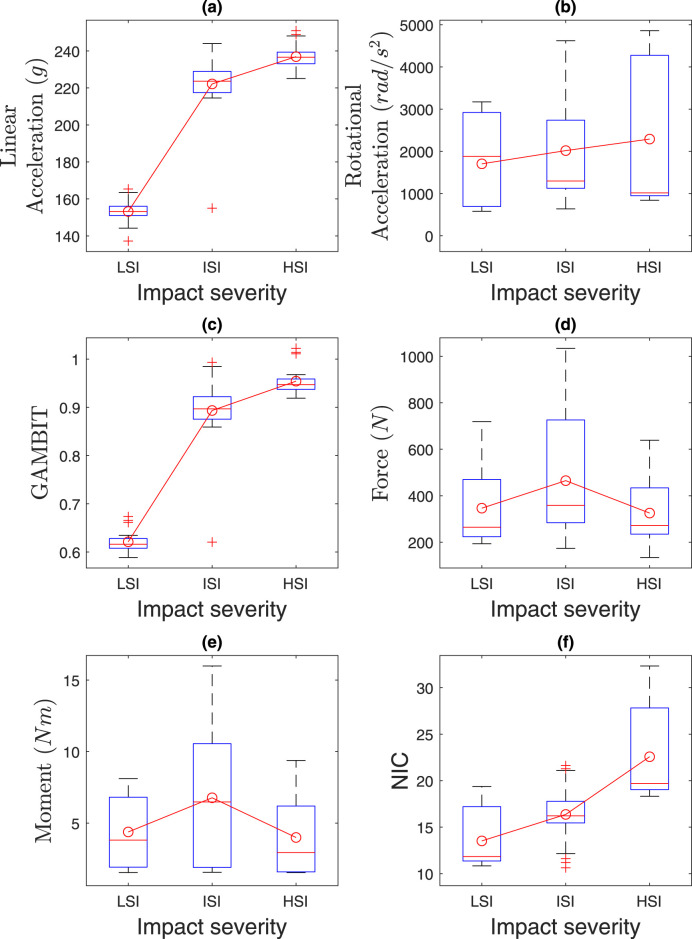
Effect of impact severities on **(a)** linear acceleration, **(b)** rotational acceleration, **(c)** GAMBIT, **(d)** neck force, **(e)** neck moment, and **(f)** NIC.

The effects of impact severities are presented in Figure 13 and Table 3. Kruskal–Wallis tests revealed significant effects of impact severities (LSI, MSI, HSI) on NIC ( p < 0.001), GAMBIT ( p < 0.001), and linear acceleration ( p < 0.001), but not on rotational acceleration ( p = 0.183), neck force ( p = 0.064), or neck moment ( p = 0.048). Pairwise Mann-Whitney U tests (Bonferroni-corrected, p < 0.0167) showed HSI significantly exceeded LSI for NIC (19.69 vs. 11.82 $m^{2}/s^{2}$ , p < 0.001), GAMBIT (0.95 vs. 0.61, p < 0.001), and linear acceleration (236.60 vs. 153.09 $m/s^{2}$ , p < 0.001), and exceeded MSI for NIC (19.69 vs. 16.21 m^2^/s^2^, p < 0.001), GAMBIT (0.95 vs. 0.90, p < 0.001), and linear acceleration (236.60 vs. 223.65 $m/s^{2}$ , p < 0.001). MSI also exceeded LSI for GAMBIT (0.90 vs. 0.61, p < 0.001) and linear acceleration (223.65 vs. 153.09 $m/s^{2}$ , p < 0.001), but not for NIC ( p = 0.030, non-significant at 0.0167).

**TABLE 3:** Effect of impact severities.

Parameter	Low severity impact (LSI)	Moderate severity impact (MSI)	High severity impact (HSI)
a	154	223	237
α	1704	2018	2,291
GAMBIT	0.62	0.89	0.95
$F_{neck}$	346	465	325
$M_{neck}$	4.38	6.77	4.29
NIC	11.51	14.38	19.28

Our model incorporates both passive and active muscle responses, as detailed in Section 2.1.2 (Equation 3), using muscle activation data from literature (Kuo et al., 2018; Kuo et al., 2019). However, in HSI, the rapid onset limits active muscle activation, making passive responses dominant. For LSI and MSI impacts, active muscle responses contribute significantly, reflecting anticipatory stiffening in automotive or sports scenarios. NIC and GAMBIT may be conservatively high due to limited active response time for HSI. However, we ignored pre-activation in all 63 cases, as it is reported that pre-activation can reduce head acceleration by ∼30% in sports contexts (Fanta et al., 2013). This was omitted to standardize responses across all impact severities. It ensures consistency across the 63 cases and avoids bias from sports-specific pre-activation data.

The average linear accelerations in Figure 13a for MSI and HSI are 1.45 and 1.54 times higher than LSI, and range from 153 g to 237 g . The maximum linear accelerations were 251 g for HSI. The average rotational accelerations in Figure 13b for MSI and HSI are respectively 1.18 and 1.35 times higher than LSI, and range from 1704 $rad/s^{2}$ to 2,290 $rad/s^{2}$ . The maximum rotational accelerations were 4859 $rad/s^{2}$ for HSI. The average GAMBIT in Figure 13c for MSI and HSI were 1.44 and 1.54 times higher than LSI. The maximum GAMBIT was 1.02 for HSI.

The neck forces and moments did not vary much based on the impact severities, as in Figures 13d,e. The NIC in Figure 13f for MSI and HSI were 1.24 and 1.68 times more than LSI. The maximum NIC value was 30.28 for HSI. In summary, the risk probability of head and neck injuries is higher for HSI and MSI than LSI.

### 4.4 Validation

The musculoskeletal model was validated in previous studies for MSI using NFL (Mortensen et al., 2018; Kuo et al., 2019; Mortensen et al., 2020) and rugby (Silvestros et al., 2024) data. However, it was not validated for LSI and HSI. To validate the model for LSI and HSI, we ran the simulation using the literature data for LSI and HSI for all neck strength. The literature only has the HIC value and max resultant linear acceleration, which we compared with our result for validation. However, the neck strengths of the experimental subjects were not mentioned in the literature. So, we ran the simulation for all neck strength. We can see from Table 4, for LSI, the experimental data indicates that the subject’s (ATD’s) neck strength was between EM 1.2 and EM 1.4. For HSI, the experimental data indicates that the subject’s (ATD’s) neck strength was between 1.4 and 1.8. The simulation results were consistent with the literature data.

**TABLE 4:** Validation for LSI and HSI data.

Data source	LSI		HSI	
	HIC	Max resultant a (g)	$HIC_{15}$	Max resultant a(g)
Experiment data	292	57	710	122
EM 1.2	303	61	740	143
EM 1.4	284	55	723	139
EM 1.8	273	53	703	133

We also compared our average results with Hybrid III ATD results (Viano, 2005) as shown in Table 5. Although the experiment design and impact forces differ, the means of most results are within one standard deviation for rotational accelerations, neck force, and moments.

**TABLE 5:** Validation of model data with ATD experiments.

LSI		
Parameter	Our study	Viano et al. (2005)
Impact force	1,210	1,051 ± 547
a	165 ± 6	24.5 ± 12.5
α	1704 ± 1,012	3,181 ± 1,343
$F_{neck}$	346 ± 170	1,486 ± 910
$M_{neck}$	4.3 ± 2.3	6.5 ± 3.4
MSI		
Impact force	1,510	2,127 ± 910
a	147 ± 17	49 ± 21
α	2017 ± 1,253	6,896 ± 2,848
$F_{neck}$	464 ± 270	1,088 ± 381
$M_{neck}$	6.7 ± 4.9	10.3 ± 6.3
HSI		
Impact force	3,455	3,107 ± 1,404
a	151 ± 6	72 ± 33
α	2,290 ± 1,672	9,306 ± 4485
$F_{neck}$	325 ± 142	855 ± 537
$M_{neck}$	7 ± 2.54	8.4 ± 6.6

From our results, the rotational accelerations of the anterolateral eccentric and posterolateral eccentric are 4.7 and 3.7 times higher than the anterior central impacts, respectively. It is mentioned in the literature that spin rotational acceleration (anterolateral eccentric or posterolateral eccentric) of the head is 3.5 times higher than whiplash acceleration (anterior impact) (Razzaghi, 2019). Our acceleration data is consistent with the literature. The distance ratio from the front of the head to the center of mass of the head and from back of the skull to the center of mass of the skull is 0.52 and 0.48. That means the anterolateral impact will create more acceleration and moment than the posterolateral impact. Viano et al. (2007) studied twenty-five helmet collisions involving NFL players and found that the maximum rotation occurred around the superior-inferior axis. The maximum acceleration was 9,678 $rad/s^{2}$ and happened on the anterolateral locations. This finding is consistent with our results.

### 4.5 Limitation

There are some limitations to this study. First, we ignored torso velocity and accelerations on the impact of head and neck injuries. We considered the torso as a fixed body. Secondly, we did not consider the whole blast impact on the head and neck injuries. We considered an idealized blast scenario, which did not account for multidimensional waveforms. This does not perfectly reproduce the experimental environment and head impacts. As a result, it may underestimate the rotational kinematics. Future models should incorporate these complexities to improve the realism. Thirdly, we consider height, weight, and anthropometric data for all tests to be the same, in order to consider the effects of neck strength, impact locations, and impact severities on head and neck injuries. However, with different anthropometric and muscle activation data, head-neck responses after an incident may vary. Also, we used the MSI muscle activation data for all cases. The muscles’ activations data were collected from (Mortensen et al., 2018; Mortensen et al., 2020).

**FIGURE 14:**
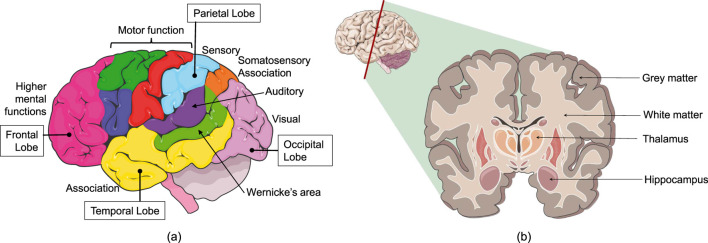
**(a)** Lobes and functional areas of the brain, **(b)** cross-section of the brain. Images modified and used with permission from Servier Medical Art, Creative Commons Attribution 4.0 Unreported License.

Fourth, while n = 3 per neck strength category provides high power (1.000 for observed GAMBIT differences, 1.000 for 20% differences, Section 2.4.3), it increases the risk of Type II errors for smaller effect sizes, particularly for non-significant parameters like rotational acceleration ( p = 0.183) and neck moment ( p = 0.048, Section 2.4). Fifth, we considered that the left and right sides of the human head and brain are symmetric. The brain has different lobes and different functional areas, as shown in Figure 14a. Also, the internal structure of brain tissue is heterogeneous and consists of grey matter, white matter, cerebrospinal fluid, and blood vessels as shown in Figure 14b. The outer layer of the brain (skull) can be considered as symmetric; however, the brain is not symmetric. Also, the functions of left-side brain and right-side brain are different. The right hemisphere is responsible for analytical thinking and logical expressions, whereas the left hemisphere is responsible for creativity and intuition. The symptoms caused by the left side impacts and right side impacts may be different. Sixth, the subjects in this study were scaled as an adult. The anthropometric data of infants, children, and adults are different. Infants are not miniature adults. Infants and children have greater head-mass to body-mass ratio. Also, their head’s center of gravity with respect to the neck is higher than that of adults (Burdi et al., 1969). All these factors may increase their TBI risk compared to adults for similar incidents (Eckner et al., 2014). The results on a scaled musculoskeletal model based on children may be different than this study and a scope for future researchers.

Also, the biomechanical model utilized in this study is based on a 50th percentile male, which may limit its generalizability to female populations. Sex-based differences in neck musculature and TBI susceptibility suggest that females may exhibit distinct injury responses due to variations in anatomy and physiology (Putra et al., 2022). This study does not quantify the differences in inter-subject variability, such as age, sex, and muscle composition. Moreover, in this study, we did not quantify the spatial distribution of impact eccentricity, such as moment arm distances. This limitation may restrict a detailed dose-response analysis that future research could explore, if relevant relationships are identified. Careful consideration should be given to the limitations outlined in this study during interpretation of the presented data and decision making. This study provides valuable insights. However, the limitations of those study should be kept in mind for any conclusion in a broader context. Further works are necessary to enhance the robustness and generalizability of the outcomes.

## 5 Conclusion

In this study, we investigated the varying relationship of neck strength, impact locations, and impact severities with head and neck injury criteria. We employed a musculoskeletal model and used forward dynamics to run 63 cases. The results were validated using different literature data. It was found that impact locations have a significant effect on head and neck injury parameters, and anterolateral eccentric impact is the riskiest impact location for both head and neck. We also found that average head and neck injury parameters do not vary more than 10% based on neck strength. Finally, the risk probability of head and neck injuries is higher for HSI and MSI than for LSI. These findings indicate that people who face an anterolateral eccentric impact are at higher risk than the person who faces an impact from other directions and should seek medical attention. The biomechanical findings of this study suggest a need for enhanced lateral impact protection in helmet design, potentially through energy-absorbing materials. These recommendations are preliminary and require clinical validation to ensure efficacy. To our knowledge, this is the first musculoskeletal model based study, where head and neck injury parameters were assessed for multiple impact locations and for all three types of severity impacts. Although the musculoskeletal-model-based framework has limitations, it provides vital information about head and neck injuries for future researchers. The high biofidelity data from this study can be utilized as the input for tissue-level and molecular-level head and neck injury studies. That will enhance our understanding about the cause-and-effects of upper extremity organ-level injuries.

## Data Availability

The raw data supporting the conclusions of this article will be made available by the authors, without undue reservation.
